# Evolutionary-new centromeres preferentially emerge within gene deserts

**DOI:** 10.1186/gb-2008-9-12-r173

**Published:** 2008-12-16

**Authors:** Mariana Lomiento, Zhaoshi Jiang, Pietro D'Addabbo, Evan E Eichler, Mariano Rocchi

**Affiliations:** 1 Department of Genetics and Microbiology, University of Bari, Via Amendola 165/A, Bari 70126, Italy; 2 Department of Genome Sciences, University of Washington School of Medicine Seattle, 1705 NE Pacific St, Seattle, WA 98195, USA; 3 Howard Hughes Medical Institute, 1705 NE Pacific St, Seattle, WA 98195, USA

## Abstract

**Background:**

Evolutionary-new centromeres (ENCs) result from the seeding of a centromere at an ectopic location along the chromosome during evolution. The novel centromere rapidly acquires the complex structure typical of eukaryote centromeres. This phenomenon has played an important role in shaping primate karyotypes. A recent study on the evolutionary-new centromere of macaque chromosome 4 (human 6) showed that the evolutionary-new centromere domain was deeply restructured, following the seeding, with respect to the corresponding human region assumed as ancestral. It was also demonstrated that the region was devoid of genes. We hypothesized that these two observations were not merely coincidental and that the absence of genes in the seeding area constituted a crucial condition for the evolutionary-new centromere fixation in the population.

**Results:**

To test our hypothesis, we characterized 14 evolutionary-new centromeres selected according to conservative criteria. Using different experimental approaches, we assessed the extent of genomic restructuring. We then determined the gene density in the ancestral domain where each evolutionary-new centromere was seeded.

**Conclusions:**

Our study suggests that restructuring of the seeding regions is an intrinsic property of novel evolutionary centromeres that could be regarded as potentially detrimental to the normal functioning of genes embedded in the region. The absence of genes, which was found to be of high statistical significance, appeared as a unique favorable scenario permissive of evolutionary-new centromere fixation in the population.

## Abstract 2

A study identifying genomic restructuring and the absence of genes as conditions permissive for the seeding of new centromeres in primates

## Background

The centromere is a complex structure ensuring the proper segregation of chromosomes in mitosis and meiosis. It usually harbors large blocks of satellite DNA (alpha satellite in primates). In spite of their complexity, centromeres have been shown to be able to relocate along the chromosome during evolution. These novel centromeres are referred to as evolutionary-new centromeres (ENCs). The first ENC examples supported by molecular cytogenetic techniques were described in non-human primates, in orthologs to human chromosome 9 [1]. Since then, several other examples have been reported in primates and other taxa [2-10]. The phenomenon implies the seeding of the novel centromere and the inactivation of the old one.

The emergence of an ENC has been hypothesized to be epigenetic in nature, that is, not accompanied by any sequence transposition. This conjectural view is supported by indirect evidence, primarily by parallels with clinical cases of human neocentromeres. These are ectopic, analphoid centromeres usually originating in chromosomal acentric fragments allowing for their mitotic survival as supernumerary chromosomes (for a review, see Marshall *et al. *[11]). They originate as opportunistic events, secondary to a chromosomal rearrangement. The latter circumstance has been regarded as strong evidence of their epigenetic nature. The detrimental phenotypic consequences of the aneuploid status frequently incurred by neocentromeres is thought to limit germline transmission and is, therefore, analogous to ENCs. Recently, however, two familial transmissions of autosomal neocentromeres, occurring in apparently normal individuals with otherwise normal karyotypes, were described [5,12]. They have been considered as ENCs at initial stages.

ENCs are relatively frequent. In macaque, for instance, 9 out of 21 centromeres are evolutionarily new [6]; in donkey at least 5 originated after a relatively short evolutionary timeframe since the donkey/zebra divergence (less than 1 million years) [8]. The relatively high number of ENCs could suggest a scenario where the absence of selective constraint allows ENC fixation. The finding, in humans, that neocentromeres do not affect gene expression [13-16] appears in line with this view.

The insight on the progression dynamics of the ENC of macaque chromosome 4 (MMU4, human 6), recently provided by Ventura *et al. *[6], has disclosed a potentially different evolutionary scenario in ENC formation. A DNA region of approximately 250 kb was pinpointed as the ENC seeding region and was shown to have been deeply affected by a variety of mutational processes, including extensive duplication on both sides of the centromere, massive insertions of small stretches of alpha-satellite DNA, and microdeletions inferred by absence of specific STS (Sequence Tagged Site) amplification. It could be supposed that this process would strongly antagonize ENC fixation because such structural variation would significantly affect the physical integrity of genes or regulatory elements located within the seeding region. Not surprisingly, Ventura *et al. *[6] observed that this region was devoid of genes. We hypothesized that this observation was not coincidental but crucial in understanding the genomic context of ENC formation.

To test this hypothesis, 14 primate ENCs were analyzed in order to: ascertain the presence of novel segmental duplications (SDs) around the ENC suggestive of a restructuring process of the kind reported by Ventura *et al. *[6]; and survey the gene density in the seeding regions. Our analysis strongly suggested that the restructuring of the neocentromeric region is an intrinsic property of ENC progression and, consequently, the highly significant absence of genes we have observed may represent a critical pre-requisite for ENC progression and fixation in the population. The 14 seeding regions were also analyzed for AT content.

## Results

### Search for evolutionary new centromeres

Published studies and our unpublished data on chromosomal evolution in primates were surveyed in the search for ENCs. We identified 31 ENCs: 15 in Catarrhini (Old World monkeys (OWMs) and Hominoidea) and 16 in Platyrrhini (New World monkeys (NWMs)). The vast majority of the NWM ENCs apparently emerged at the breakpoint of a chromosomal fission or repositioned from a telomere to the other telomere (see, for instance, the evolution of chromosome 3 [5]). Centromeres of human acrocentrics 15 and 14 are examples of ENCs that originated at a breakpoint and at a telomere, respectively, following a chromosomal fission [3]. Their short arms consist of several megabases of acquired sequences. These circumstances suggested that telomeric ENCs could represent a different ENC category, with different progression dynamics. We therefore excluded these ENCs from the analysis and focused our investigation on the ENCs that emerged inside a chromosome and were not concomitant to a disruption of the seeding region.

Fourteen ENCs met these conservative criteria: one in woolly monkey (*Lagothrix lagothricha*, LLA, Atelinae, NWM), eight in OWMs [6], one in white-cheeked gibbon (*Nomascus leucogenys*, NLE) [17], one in orangutan (*Pongo pygmaeus*, PPY) [18], and three in humans (*Homo sapiens*, HSA) [5,18,19]. The ENC that emerged on chromosome 7 (human 8) of woolly monkey (NWM) has not been previously published. The evolutionary history of chromosome 8, supporting the emergence of an ENC in this primate, is summarized in Additional data file 1; fluorescence *in situ *hybridization (FISH) examples shown given in Additional data file 2a, b. Bacterial artificial chromosome (BAC) clones used in the analysis are reported in Additional data file 3. The eight ENCs found in macaque (Cercopithecinae) are also present in the silvered leaf monkey (*Trachypithecus cristatus*, TCR, Colobinae), indicating that all ENCs originated in the Cercopithecinae/Colobinae common ancestor. The rhesus macaque was used as a representative of OWMs because its genome has been fully sequenced [20].

Reiterative FISH experiments with corresponding human BAC clones were performed in non-human primate metaphases in order to precisely map these ENCs on the human sequence used as a reference (build35 assembly, March 2004; an example is shown in Additional data file 2c). The macaque sequence was used as a reference for the three human ENCs (rheMac2 release, January 2006). The position of the human ENCs in macaque was defined using macaque BAC clones hybridized to human metaphases. The results are summarized in Table 1. In some cases a BAC generated split signals on both sides of the centromere (Table 1, entries in bold), while flanking BACs gave a single signal on the expected pericentromeric side. The sequence corresponding to the splitting BAC was flagged as the ENC seeding region. In other cases the position of the ENC was defined by two overlapping BACs mapping on opposite sides of the ENC.

**Table 1:** Definition of the ENC seeding region in the reference genome

Chromosome	ENC position	Size (kb)	p arm BAC	Position in HSA (hg17) or MMU (rheMac2)	q arm BAC	Position in HSA (hg17) or MMU (rheMac2)	AT content (%)
**Platirrhini**							
LLA7 (HSA8)	Chr8:63,002,317-63,047,396	45	RP11-953L16	Chr8:62,816,386-63,002,317	RP11-159F22	Chr8:63,047,396-63,204,407	63.9
							
**Catarrhini**							
MMU2 (HSA3)	Chr3:164,221,008-164,539,729	319	RP11-449O23	Chr3:164,054,860-164,221,008	RP11-418B12	Chr3:164,539,729-164,707,135	65.9
MMU4 (HSA6)	Chr6:145,651,644-145,845,896	194	**RP11-474A9**	Chr6:145,651,644-145,845,896			63.2
MMU12 (HSA2q)	Chr2:138,847,788-138,947,383	99	RP11-343I5	Chr2:138,777,146-138,947,383	RP11-846E22	Chr2:138,847,788-139,025,935	63.1
MMU13 (HSA2p)	Chr2:86,680,785-86,885,407	204	**RP11-722G17**	Chr2:86,680,785-86,885,407			60.0
MMU14 (HSA11)	Chr11:5,856,181-5,864,725	8	RP11-625D10	Chr11:5,667,339-5,864,725	RP11-661M13	Chr11:5,856,181-6,043,020	62.8
MMU15 (HSA9)	Chr9:122,486,836-122,532,865	46	RP11-64P14	Chr9:122,344,545-122,532,865	RP11-1069J21	Chr9:122,486,836-122,680,563	62.4
MMU17 (HSA13)	Chr13:61,178,154-62,520,878	1,343	***RP11-543A19***	Chr13:61,111,769-61,178,154	***RP11-527N12***	Chr13:62,520,878-62,699,203	66.2
MMU18 (HSA18)	Chr18:50,313,129-50,360,135	47	RP11-61D1	Chr18:50,155,761-50,313,129	RP11-289E15	Chr18:50,360,135-50,526,341	gap
NLE15 (HSA11)	Chr11:89,446,995-89,488,776	42	RP11-529A4	Chr11:89,286,313-89,446,995	RP11-1129K7	Chr11:89,488,776-89,644,713	63.8
PPY11 (HSA11)	Chr11:20,180,424-20,332,556	152	**RP11-56J22**	Chr11:20,180,424-20,332,556			61.2
							
**HSA**							
HSA3 (MMU2)	Chr2:14,301,434-14,386,749	85	CH250-111O10	Chr2:14,301,466-14,396,994	CH250-4J18	Chr2:14,386,749-14,533,296	62.3
HSA6 (MMU4)	Chr4: 57,710,481-57,863,274	153	**CH250-20M17**	Chr4: 57,710,481-57,863,274			66.1
HSA11 (MMU14)	Chr14:17,109,970-17,281,610	171	CH250-111J7	Chr14:17,015,710-17,109,970	CH250-37N19	Chr14:17,281,610-17,299,898	63.4

Seeding regions of the studied ENCs, defined by a splitting BAC (in bold) or by overlapping BACs mapping in opposite sides of the centromere (p arm/q arm). In the latter case the overlapping portion of the two BACs was assumed as the seeding point. In MMU17 (human 13), several contiguous human BACs gave split signals. The table lists the most external ones (in italics). The human genome was used as a reference genome for non-human primate ENCs. The macaque genome was used as a reference for the three human ENCs (see text).

### Ancestral organization of regions where ENCs were seeded

The human regions orthologous to the sequence domains where the non-human ENCs were seeded were investigated for evolutionary conservation against mouse and dog genomes by visually inspecting the University of California Santa Cruz (UCSC) Comparative Genomics Net tracks [21]. The analysis was performed in order to validate the human sequence as *bona fide *reference sequence with respect to the changes the ENC regions underwent during evolution. We performed a similar comparative analysis for macaque regions corresponding to the three human ENCs for which the macaque was used as a reference. In both human and macaque sequences, the analysis encompassed approximately 2 Mb on each side of the seeding point. Substantial differences were found only in mouse (breaks or inversions at regions corresponding to human chromosome 2 (85.7-86.7 Mb and 137.6-137.7 Mb), chromosome 8 (61.9-62.8 Mb), and chromosome 11 (88.4-89.2 Mb)). No rearrangements were found in the dog, with the exception of the cluster of olfactory receptor (OR) genes located at 121.5-122.3 Mb in human chromosome 9 and absent in dog. The human/dog concordance strongly suggests that these rearrangements are derivative in mouse.

### Tempo of evolutionary-new centromere seeding

**Figure 1:**
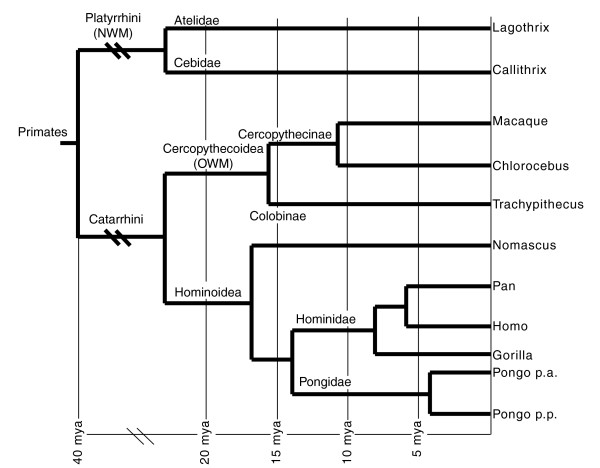
**The phylogenetic relationships of the species under study**. Data on OWMs and Hominoidea are from Raaum *et al. *[22], while those on NWMs are from Schneider *et al. *[24].

As mentioned, all eight ENCs found in OWMs were present in both macaque (Cercopithecinae) and silvered leaf monkey (Colobinae) species. Therefore, all originated before the Cercopithecinae/Colobinae divergence (see Figure 1), estimated to have occurred 16 million years ago (mya) [22]. The position of the centromere on chromosomes orthologous to HSA2q (MMU12), HSA13 (MMU17), and HSA18 (MMU18) is shared by Hominoidea and NWMs [23] (unpublished data). The ENC seeding on these chromosomes, therefore, occurred in OWMs (Cercopithecoidea) after their divergence from Hominoidea (Figure 1), which was approximately 23 mya [22]. It was not possible to precisely define the upper temporal limit of the remaining OWM ENCs because the position of the centromere on orthologous NWM and Hominoidea chromosomes showed discrepancies [1,5,19].

The ENC on orangutan chromosome 11 is *Pongo*-specific [18] and is shared by both orangutan subspecies (*Pongo pygmaeus abelii *and *Pongo pygmaeus pygmaeus*). Consequently, it was seeded within the interval 4-14 mya (between Pongidae/Hominidae and PPY *abelii*/PPY *pygmaeus *splits, respectively). The HSA11 ENC is, very likely, *Hominidae*-specific [18]. Thus, it dates within the interval 8-14 mya (after Pongidae/Hominidae split and before gorilla-pan-homo divergence, respectively). HSA3 and HSA6 ENCs are shared by great apes, so they date prior to 8 mya. Uncertainty on the ancestral position of the centromere in these chromosomes impinges on the uncertainty of the upper temporal limit of their occurrence [5,19]. For the ENC of the woolly monkey (LLA7, NWM, Atelidae), we could define only the upper temporal limit of 22-23 mya, which is the estimated divergence time of the Atelidae (LLA) and Cebidae (CJA) lineages [24].

### Search for segmental duplications around evolutionary-new centromeres

SD analysis was straightforward for the three human ENCs (chromosomes 3, 6 and 11) due to the high quality of the sequence assembly within these human pericentromeric regions [25]. Duplications were found in the pericentromeric regions of all three human chromosomes. On chromosome 6 and particularly on chromosome 3, intrachromosomal duplications predominate. The duplication status of the sequenced macaque and orangutan genomes is less accurate with respect to humans because of the severe limitations intrinsic to the whole genome shotgun (WGS) sequencing assembly approach [26] in resolving high-identity duplications (note that whole genome sequence data are not currently available for the white-cheeked gibbon and woolly monkey).

To circumvent, at least in part, these problems, we exploited complementary bioinformatic and molecular cytogenetic techniques because they are partially 'assembly independent'. First, we examined each of the ENC regions for the presence of recent duplications in various primates using WGS sequence detection (WSSD) [27], where whole genome shotgun (WGS) reads from each primate are mapped against the human reference genome (hg17). Table 2 lists WSSD positive intervals detected for each primate species. Segmental duplications were detected, for example, on MMU4 (HSA6), MMU17 (HSA13), and PPY11 (HSA11). We then selected and tested various BAC clones by FISH. Some duplication data already resulted from experiments aimed at identifying the seeding region using human BAC clones (see above). However, split signals on both sides of the centromere could be alternatively interpreted as due to a disruption of distinct, non-duplicated sequences composing the human BAC, as a consequence of the colonization of alpha satellite DNA. Additionally, orthologous human clones may not be suitable for the analysis because of the restructuring process that could have substantially altered the pericentromeric sequences within each species. Final, new material, not represented in human BACs, may exist within these locations due to lineage-specific interchromosomal duplications.

**Table 2:** Duplication analyses in ENC regions

						Non-redundant WSSD base pair (bp)			
						
ENC	Start (HAS hg17)	Start+1M	End (HS A hg17)	End+1M	Size	HSA	PTR	PPY	MMU
**Narrow interval**									
MMU2 (HSA3)	164,221,008		164,539,729		318,722	0	0	0	0
MMU4 (HSA6)	145,651,644		145,845,896		194,253	0	0	0	104,409
MMU12 (HSA2)	138,847,788		138,947,383		99,596	0	0	0	0
MMU13 (HSA2)	86,680,785		86,885,407		204,623	24,002	0	0	0
MMU14 (HSA11)	5,856,181		5,864,725		8,545	0	0	0	0
MMU15 (HSA9)	122,486,836		122,532,865		46,030	0	0	0	0
MMU17 (HSA13)	61,178,154		62,520,878		1,342,725	24,879	15,879	103,912	85,133
MMU18 (HSA18)	50,313,129		50,360,135		47,007	0	0	0	0
PPY11 (HSA11)	20,180,424		20,332,556		152,133	0	0	126,135	0
									
**Larger interval**									
MMU2 (HSA3)		163,221,008		165,539,729	2,318,722	0	0	0	24,053
MMU4 (HSA6)		144,651,644		146,845,896	2,194,253	0	0	0	115,053
MMU12 (HSA2)		137,847,788		139,947,383	2,099,596	0	0	17,001	1,706
MMU13 (HSA2)		85,680,785		87,885,407	2,204,623	1,227,738	309,321	0	19,317
MMU14 (HSA11)		4,856,181		6,864,725	2,008,545	0	0	13,379	0
MMU15 (HSA9)		121,486,836		123,532,865	2,046,030	0	0	0	0
MMU17 (HSA13)		60,178,154		63,520,878	3,342,725	160,4637	98,004	144,056	85,133
MMU18 (HSA18)		49,313,129		51,360,135	2,047,007	0	0	0	0
PPY11 (HSA11)		19,180,424		21,332,556	2,152,133	0	0	784,808	0

We estimated the number of duplicated base-pairs predicted in each of the ENC intervals using the WSSD method; duplications >10 kb and >94% were detected with the exception of the macaque, where a threshold of >88% was used due to the greater sequence divergence of the human and macaque genomes. The analysis was performed separately for each of the four primate species. Two different ENC intervals were considered: a narrow interval, as defined in Table 1 (upper dataset) and a larger interval adding 1 Mbp to each side of the region (lower dataset).

**Figure 2:**
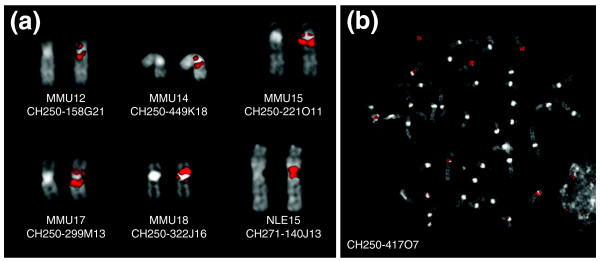
**FISH examples**. **(a) **Examples of FISH experiments using species-specific BAC clones yielding duplicated signals around the centromere. The CH250 and CH271 are BAC libraries specific for macaque and gibbon, respectively. The DAPI-stained chromosome without the signal is reported on the left to better show the morphology of the chromosome. **(b) **FISH experiment using the BAC clone CH250-417O7 (MMU2) on a macaque metaphase, showing pericentromeric signals on several chromosomes.

Considering these potential biases, we also selected species-specific BAC clones identified with different approaches. For macaque we took advantage of the data on MMU BAC clones available at the Bioinformatics Research Laboratory of the Baylor College of Medicine, Houston, TX, USA [28]. For orangutan (PPY) and white-checked gibbon (NLE), we queried appropriate BAC-end sequences from CHORI-276 (PPY) and CHORI-271 (NLE) BAC libraries using the Trace Archive database of the NCBI [29]. A BAC library was not available for the woolly monkey (LLA). The phylogenetic distance of this NWM species coupled with the potential degenerative consequences of pericentromeric restructuring processes prompted us to discard the woolly monkey from the pericentromeric duplication analysis. Relevant FISH results of species-specific BAC clones yielding duplicated signals around the ENCs are reported in Table 3 (all tested clones are given in Additional data file 4); examples are shown in Figure 2 and Additional data file 2e, f. We discovered pericentromeric duplications mapping near the centromeres for almost all ENCs. One BAC-end of asterisked BACs in Table 3 and Additional data file 4 was identified by RepeatMasker as entirely composed of 171 bp alpha satellite repeats. No internal repeat was found truncated, and the homology with the apha satellite consensus ranged from 75% to 90%.

**Table 3:** Species-specific BACs yielding duplicated signals oround ENCs

ENC	BAC	Position in HSA (May 2004)
MMU13 (HSA2p)	CH250-565F19*	Chr2:86,755,212-alphoid
	CH250-417O7	Chr2:86,785,727-repeat
	CH250-371E19*	Chr2:86,870,586-alphoid
		
MMU12 (HSA2q)	CH250-359C1	Chr2:138,344,201-138,510,183
	CH250-158G21	Chr2:138,478,651-138,621,067
	CH250-18F12*	Chr2:138,643,711-alphoid
		
MMU14 (HSA11)	CH250-444O7*	Chr11:5,861,684-alphoid
	CH250-499K18*	Chr11:6,038,164-alphoid
		
MMU15 (HSA9)	CH250-221O11*	Chr9:122,220,400-alphoid
		
MMU17 (HSA13)	CH250-310C22	Chr13:61,479,136-61,591,608
	CH250-299M13	Chr13:61,503,914-61,617,441
	CH250-115C9	Chr13:61,540,997-61,676,877
		
MMU18 (HSA18)	CH250-322J6	Chr18:50,437,322-repeat
		
NLE15 (HSA11)	CH271-140J13	Chr11:89,572,864-repeat

Species-specific BAC clones yielding duplicated signals around the ENC. Their specific pericentromeric location, confirmed by FISH, was derived by their BAC-end(s) mapping. *One BAC-end of these BACs is entirely composed of alphoid repeats. The FISH signal, however, was not centromeric, indicating that the alphoid content of the BAC was marginal. See Figure 1 for examples.

Two findings were of particular interest. Four nearly overlapping human BACs (RP11-543A19, -1043D14, -539I23, and -527N12) covering a region of 1.3 Mb (chr13: 61,111,769-62,699,203) around the MMU17 ENC gave duplicated signals around the centromere. Additionally, the two human BACs defining the ENC of MMU2 (HSA3) are 319 kb apart (Table 1). Three BACs spanning this interval (RP11-1089F10, -1142P11, and -10O22) failed to give any FISH signals in macaque, suggesting a deletion of the corresponding region within the macaque lineage. To exclude the possibility of a technical artifact, we mixed on the same slide human and macaque metaphases, added an excess of probe, and extended the hybridization time for three days. Again in these conditions, no signal was detected in macaque metaphases, while strong signals were present in human metaphases. We performed a BLAST sequence similarity using the human 319 kb region as query against macaque sequences deposited in the Trace Archive database [29]. Only very small stretches (less than 1 kb) of homologous DNA were found externally located with respect to a central chr3:164,271,000-164,461,000 region (190 kb) in which no homology was detected (data not shown). Additionally, the macaque BAC clone CH250-91J4, identified at the Baylor College database (see above), mapping at HSA chr3:164,777,357-164,967,209, which is slightly external to the 'deleted' region, failed to yield any signal in human metaphases (data not shown). Altogether, these data strongly suggest that the region is highly rearranged in macaque.

### Gene content at evolutionary-new centromere regions

**Figure 3:**
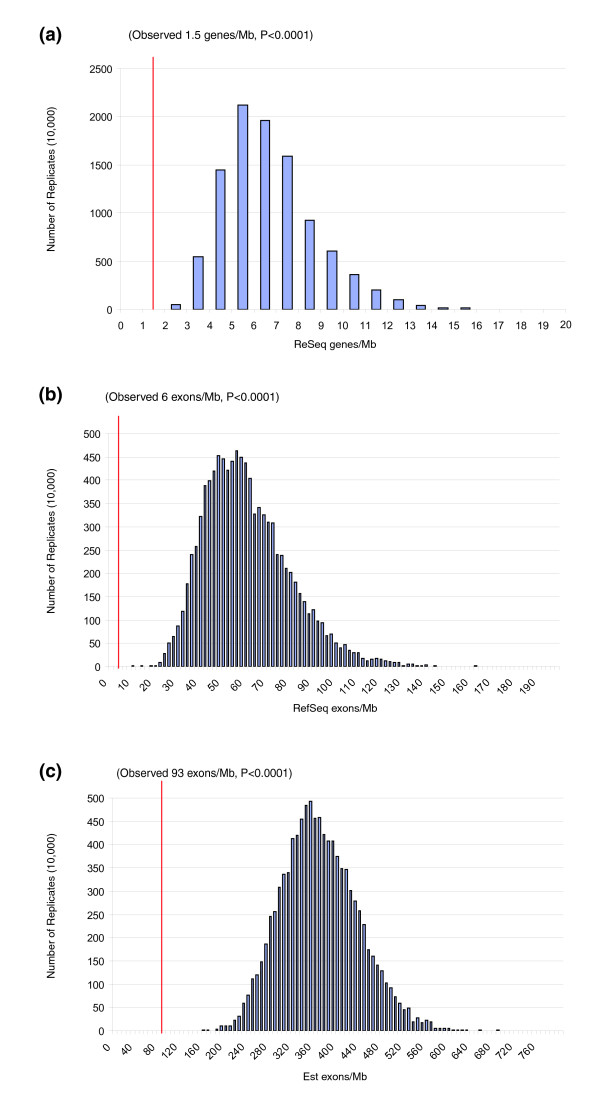
**Gene density simulations**. The observed density of **(a) **genes (Refseq), **(b) **Refseq exons and **(c) **expressed sequence tag (Est) exons within the corresponding region of the 14 ENCs were compared against a simulated set of 10,000 regions distributed randomly within the human genome (see Materials and methods). A significant depletion of exons and genes was observed.

We carefully analyzed the human genome (used as reference for non-human ENCs) and the macaque genome (used as reference for the three human ENCs) for annotated genes mapping within, or in proximity of, ENC seeding regions. The analysis was performed by querying the human and macaque RefSeq-related tracks of the UCSC genome browser [21] (hg17 assembly, RheMac2 assembly). No RefSeq genes were identified within the seeding regions as defined above (Table 1). In order to assess the statistical significance of gene depletion in the regions where ENCs were seeded, we performed a gene/exon density simulation (see Materials and methods) for 14 ENC regions. We found that the gene/exon density of the 14 ENCs is significantly depleted (*p *< 0.0001) when compared to random simulated data (Figure 3a-c). Table 4 reports the most proximal and distal RefSeq genes with respect to the ENC seeding point. The distance between the two genes is reported in the second column. Clusters of olfactory receptor genes flank the ENCs of chromosomes MMU14 (HSA11), MMU15 (HSA9), and HSA11 (MMU14). These OR clusters were not considered because OR genes are extremely redundant and a large number of these are pseudogenic within the primate lineage. The inactivation of a few of them would unlikely have strong phenotypical consequences. It is worth noting, in this context, that more than half of OR genes became inactive in recent human evolution [30].

**Table 4:** RefSeq genes flanking the ENCs

ENC	Interval (Mb)	Left gene	Position in HSA (hg17) or MMU (rheMac2)	Right gene	Position in HSA (hg17) or MMU (rheMac2)
**Platirrhini**					
LLA7 (HSA8)	0.534	ASPH	Chr8:62,699,652-62,789,681	FAM77D	Chr8:63,324,055-64,074,765
					
**Catarrhini**					
MMU2 (HSA3)	3.607	C3orf57	Chr3:162,545,283-162,572,573	SI	Chr3:166,179,388-166,278,984
MMU4 (HSA6)	0.772	UTRN	Chr6:144,654,566-145,215,861	EPM2A	Chr6:145,988,141-146,098,684
MMU13 (HSA2p)	0.097	RNF103	Chr2:86,742,174-86,762,636	RMD5A	Chr2:86,859,351-86,914,090
MMU14 (HSA11)	1.213	MMP26	Chr11:4,966,000-4,970,233	C11orf42	Chr11:6,183,374-6,188,935
MMU15 (HSA9)	0.423	PTGS1	Chr9:122,212,783-122,237,535	PDCL	Chr9:122,660,178-122,670,394
MMU12 (HSA2q)	0.485	HNMT	Chr2:138,555,540-138,607,665	LOC339745	Chr2:139,093,103-139,164,532
MMU17 (HSA13)	4.888	PCDH20	Chr13:60,881,822-60,887,282	PCDH9	Chr13:65,774,968-66,702,464
MMU18 (HSA18)	0.247	C18orf54	Chr18:50,139,169-50,162,379	C18orf26	Chr18:50,409,388-50,417,722
NLE14 (HSA11)	2.746	CHORDC1	Chr11:89,574,265-89,595,854	MTNR1B	Chr11:92,342,437-92,355,596
PPY11 (HSA11)	0.203	DBX1	Chr11:20,134,336-20,138,446	HTATIP2	Chr11:20,341,924-20,361,904
					
**HSA**					
HSA3 (MMU2)	0.641	EPHA3 in HSA (not annotated in	Chr3:89,239,364-89,613,972	PROS1 (L31380 in MMU)	Chr3:95,074,647-95,175,395
		MMU)	(MMU2:13,335,593-13,694,578)		(Chr2:14,335,824-14,391,596)
HSA6 (MMU4)	0.897	PRIM2A in HAS (not annotated in	Chr6:57,290,381-57,621,334	KHDRBS2 in HAS (not annotated in	Chr6:62,447,824-63,054,091
		MMU)	(2 dup in MMU:	MMU)	(3 dup in MMU:
			MMU4:56,935,673-57,245,600		Chr4:58,142,819-58,698,705
			MMU11:20,043,342-20,044,345)		Chr17:3,473,312-3,473,395
					Chr8:138,072,498-138,196,040)
HSA11 (MMU14)	1.280	LRRC55 in HAS (not annotated in	Chr11:56,705,797-56,714,154	PTPRJ in HAS (not annotated in MMU)	Chr11:47,958,689-48,146,246
		MMU)	(MMU14:16,226,175-16,234,557)		(Chr14:23,931,871-24,124,487)

Position of the most proximal and distal genes with respect to each ENC seeding region, calculated in the reference genome (see text). The interval size, in Mb, between the two genes is reported in column 2.

### AT content

The precise location of some human neocentromeres has been achieved through CENP-A mapping by chromatin immunoprecipitation (ChIP)-on-chip experiments (reviewed by Marshall *et al. *[11]). AT content has been shown to be one of the few common features shared by these neocentromeres. We calculated the AT content for the human domains corresponding to the ENC seeding regions as defined in Table 1. The results are reported in the last column of Table 1.

## Discussion

The organization, evolution and function of eukaryotic centromeres represent a deficiency in our understanding of genome biology. The discovery of human clinical neocentromeres and ENCs has further complicated, on one hand, our understanding of the centromere. On the other hand, neocentromeres and ENCs have allowed an initial dissection of centromere complexity. They have made evident, for instance, its epigenetic nature. The ENC analysis we have accomplished in the present study has contributed to the identification of factors that, very likely, play a crucial role in ENC progression and fixation in the population. We have provided strong evidence that the pericentromeric duplication activity is an intrinsic property of ENCs. This conclusion was mainly supported by FISH experiments using species-specific BAC clones that detected SDs around the centromere in almost all studied ENCs. A deep restructuring was particularly evident in MMU17 (human 13) and MMU2 (human 3). The latter ENC showed a large deletion. This observation is not unexpected and could be generated by allelic non-homologous recombination occurring in one side of the centromere. Our overall results indicate that deep restructuring is a feature inherent to pericentromeric duplication activity triggered by the ENCs. Our analysis also indicated that species-specific probes are the most appropriate for detecting potential interchromosomal duplications (see ENCs of MMU12, 13, 14, 15 and 17).

Contrary to what we detected in the ENC of MMU4 (human 6), where SDs were strictly intrachromosomal [6], we found that SDs associated with other ENCs were both inter- and intrachromosomal (for example, Figure 2b). Pericentromeric analysis in humans has indicated that the majority of SDs are interchromosomal. It could be hypothesized that intrachromosomal duplications arose first, followed by interchromosomal ones. This interpretation, however, clashes with the finding, in humans, that the interchromosomal versus intrachromosomal SD ratio usually increases approaching the centromere, with the exception of few chromosomes [31]. Interestingly, three of these exceptions (chromosomes 3, 6 and, partially, 11) correspond to ENCs. It can be hypothesized that these differences could be a reflection of the age of the ENCs. Intrachromosomal SDs occur first but then as centromeres become established they begin to exchange between non-homologous chromosomes, such that eventually interchromosomal duplications outnumber the intrachromosomal.

Studies on selected human neocentromeres have shown that the chromatin remodeling accompanying the neocentromere seeding does not alter gene expression [13-16]. By analogy with ENCs, the presence of genes would not negatively affect, *per se*, ENC function. Our studies suggest that the subsequent duplication activity, implying deep restructuring, would, on the contrary, antagonize ENC fixation. In this scenario, the only condition compatible with ENC fixation in the population would be either the lack of genes in the ENC seeding region or the presence of multi-copy gene family where loss would be tolerated. The study provided strong support for this scenario: the ENC seeding regions we have examined are significantly depleted of genes. The MMU17 (HSA13) ENC is of relevance in this context. It exhibits the largest gene desert (4.9 Mb) and one of the largest duplicated regions (1.3 Mb). The non-casual matching is further reinforced by the analysis of the pattern of SDs around this repositioned centromere in three distinct regions showing large-scale variation in OWM species as reported by Cardone *et al. *[23]. This extensive variation could be interpreted as further evidence of relaxed constraint on duplication activity due to the large size of the gene desert.

In an individual heterozygous for an ENC, a meiotic exchange within the region delimited by the old and the novel centromeres would produce dicentric and acentric chromosomes, mimicking the consequences of a pericentric inversion. These events are supposed to affect the fitness of heterozygous carriers negatively. Meiotic drive in females in favor of the repositioned chromosome is a possible explanation, as reported for Robertsonian fusion in humans [32]. Genetic drift and population structure can also be hypothesized to have played an important role in neocentromere fixation.

The AT content of all gene deserts flanking the ENCs was higher than 59%, that is, the average of the entire human genome [33] (see last column of Table 1). These findings, however, could just reflect the high AT content of gene-poor regions.

## Conclusion

Our study strongly supports the hypothesis that the evolutionary fate of a repositioned centromere is largely dependent upon a low gene density of the seeding region. This feature appears to be the consequence of the peculiar dynamics of ENC progression associated with extensive restructuring of the region, including deletions, that can be assumed as potentially detrimental in genic regions of the genome.

## Materials and methods

### Cell lines

Metaphase preparations were obtained from cell lines (lymphoblasts or fibroblasts) from the following ape species: common chimpanzee (*Pan troglodytes*, PTR), gorilla (*Gorilla gorilla*, GGO), Bornean orangutan (*Pongo pygmaeus pygmaeus*, PPY), white-cheeked gibbon (*Nomascus leucogenys*, NLE). OWMs: rhesus monkey (*Macaca mulatta*, MMU), vervet monkey (*Chlorocebus aethiops*, CAE, Cercopithecinae), silvered leaf monkey (*Trachypithecus cristatus*, TCR, Colobinae). NWMs: wooly monkey (*Lagothrix lagothricha*, LLA, Atelidae), common marmoset (*Callithrix jacchus*, CJA, Callitricidae).

### FISH experiments

DNA extraction from BACs was reported previously [2]. Co-hybridization FISH experiments were performed essentially as described by Lichter *et al. *[34]. To suppress cross-hybridization signals due to repeat sequences, COT1 DNA (5 μg) was added to the hybridization mixture. Digital images were obtained using a Leica DMRXA2 epifluorescence microscope equipped with a cooled CCD camera (Princeton Instruments, Princeton, NJ, USA). Cy3-dUTP, Fluorescein-dCTP, Cy5-dCTP and DAPI fluorescence signals, detected with specific filters, were recorded separately as grayscale images. Pseudocoloring and merging of images were performed using Adobe Photoshop™ software.

### BAC-end sequence analysis

BAC-end sequences were retrieved from the Trace Archive database [29]. They were then analyzed using the RepeatMasker software [35] in order to identify BAC-ends partially or entirely composed of repeat sequences. The software provides information on the extension and type of repeat.

### Primate segmental duplication characterization in ENC regions

In order to identify segmental duplication content in various primates, we used the previously described assembly-independent approach (WSSD) where WGS sequence reads [27] from each query primate genome were mapped against regions from the human genome reference sequence (build35) corresponding to the ENCs. We considered regions of excess WGS read depth (≥ mean + 1.5 × standard deviation) to represent putative duplicated regions in each primate. Due to different genomic sequence divergences between each primate and the human reference sequence, we used sequence identity thresholds of ≥ 88% to map macaque reads while ≥ 94% was used for alignment of reads from chimpanzee and orangutan.

### Gene/exon density simulation

In order to statistically assess the depletion of gene/exon density in the regions where ENCs were seeded, we performed the gene/exon density simulation as follows. First, we computed the average gene/exon density for the 14 ENC regions based on their annotation within the human genome. This became our observed value for gene/exon density within ENC regions (red line in Figure 3). Next, we randomly selected the same number of gap-free base-pairs (23.2 Mbp) from the human genome and computed the average gene/exon density for these randomly selected intervals. We generated 10,000 replicates and plotted the distribution of gene/exon density based on this simulation. We computed an empirical *p*-value as the number of replicates with gene/exon density equal to or lower than the observed density in 10,000 replicates. We repeated the analysis excluding ENCs that had been identified within the human lineage of evolution (n = 3) and obtained similar results (data not shown). For genes, we considered the position of all human non-redundant genes (RefSeq gene n = 22,589) and their corresponding exons as determined by the UCSC genome browser [21]. As a second analysis to assess transcript density, we independently mapped the location of all spliced human ESTs (n = 4,246,559) to the human genome (build35) and selected the location of the highest alignment score. If an EST/transcript mapped to two or more locations with an equivalent score, one was selected at random, such that each transcript was assigned once and only once to the human genome. As part of this analysis, we clustered exons that overlapped as a result of alternative splicing and counted each cluster as a single exon.

## Abbreviations

BAC: bacterial artificial chromosome; ChIP: chromatin immunoprecipitation; ENC: evolutionary-new centromere; FISH: fluorescence *in situ *hybridization; mya, million years ago; NWM: New World monkey; OR: olfactory receptor; OWM: Old World monkey; SD: segmental duplication; UCSC: University California Santa Cruz; WGS: whole genome shotgun; WSSD: WGS sequence detection.

## Authors' contributions

ML planned and carried out the molecular cytogenetic experiments; PD analyzed the bioinformatic data; ZJ and EEE performed the statystical analysis; MR designed and coordinated the study and wrote the paper. All authors read and approved the final manuscript.

## Additional data files

The following additional data are available with the online version of thispaper. Additional data file 1 illustrates the evolutionary history of chromosome 8 in primates. Additional data file 2 provides examples of FISH experiments. Additional data file 3 lists the human probes used to track the evolutionary history of chromosome 8. Additional data file 4 lists the species-specific BAC clones used in FISH experiments to detect pericentromeric segmental duplications.

## Supplementary Material

Additional data file 1 Evolutionary history of chromosome 8 in primates. Click here for file

Additional data file 2 Examples of FISH experiments. Click here for file

Additional data file 3 Human probes used to track the evolutionary history of chromosome 8. Click here for file

Additional data file 4 Species-specific BAC clones used in FISH experiments to detect pericentromeric segmental duplications. Click here for file
