# Supplementary material for: Evolutionary-new centromeres preferentially emerge within gene deserts
Source: Genome Biol. 2008 Dec 16;9(12):R173. doi: 10.1186/gb-2008-9-12-r173 (PMC2646277; doi:10.1186/gb-2008-9-12-r173)
Supplement: Additional data file 1 — Evolutionary history of chromosome 8 in primates. [file gb-2008-9-12-r173-S1.pdf]

Supplemental Figure 1

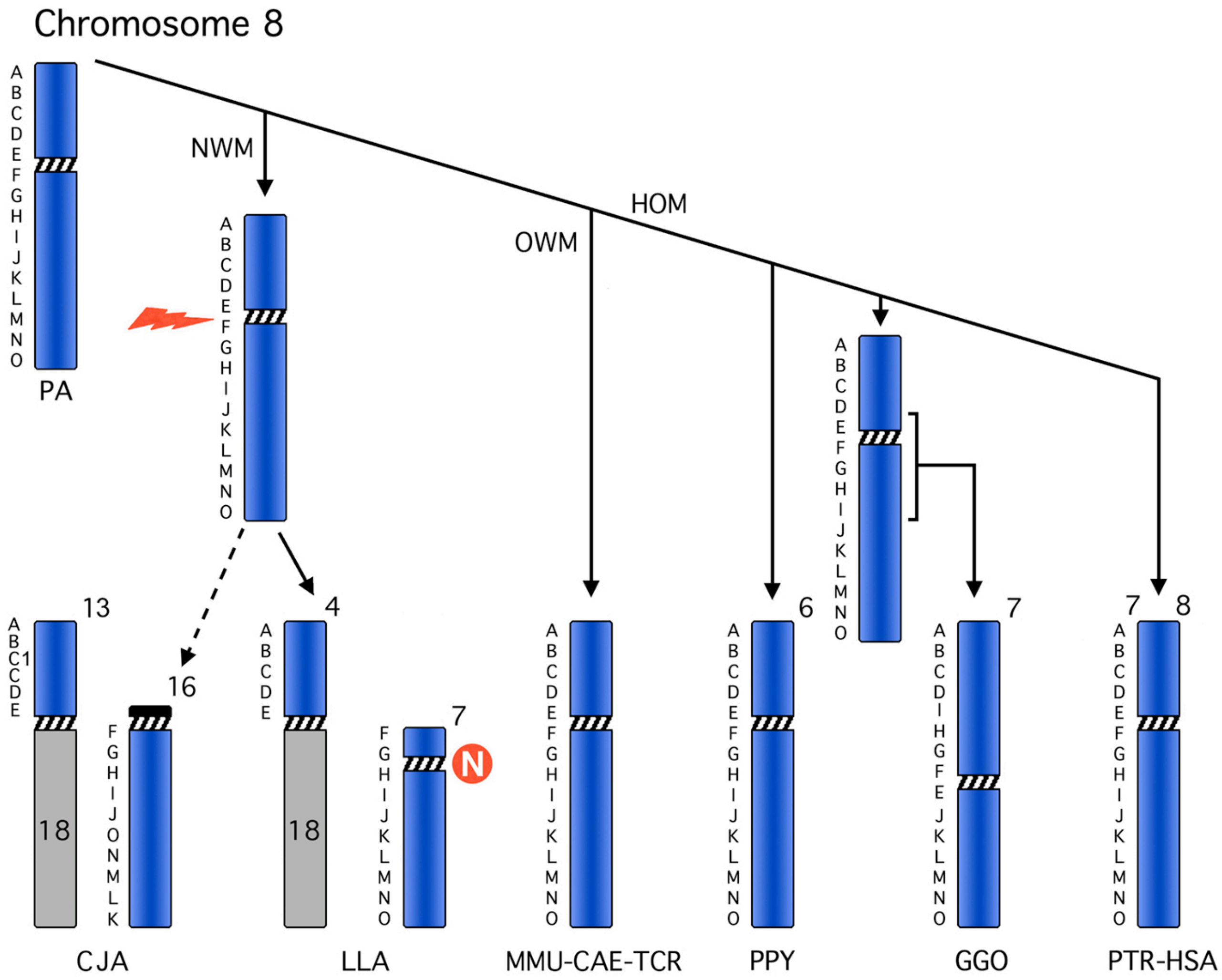

The figure illustrates the evolutionary history of chromosome 8 in primates. The black letters on the left of each chromosome refer to human BAC clones, reported in Supplemental Table 1, used in FISH experiments to assess the marker order arrangement of each species. The chromosome number is reported on top of each chromosome. Rearrangements and the hypothesized marker order of Primate Ancestor (PA) are proposed in a maximum parsimony framework. Markers arrangements in woolly monkey (LLA) chromosome 7 strongly support the emergence of an ENC ("N" in the red circle). The rearrangements that lead to the CJA form are not shown. HOM = Hominoidea; PTR = *Pan troglodytes*; GGO = *Gorilla gorilla*; PPY = *Pongo pygmaeus*; MMU = *Macaca mulatta*; CAE = *Chlorocebus aethiops*; TCR = *Trachypithecus cristatus*; LLA = *Lagothrix lagothricha*; CJA = *Callithrix jacchus*.
