# Supplementary material for: Evolutionary-new centromeres preferentially emerge within gene deserts
Source: Genome Biol. 2008 Dec 16;9(12):R173. doi: 10.1186/gb-2008-9-12-r173 (PMC2646277; doi:10.1186/gb-2008-9-12-r173)
Supplement: Additional data file 2 — Examples of FISH experiments. [file gb-2008-9-12-r173-S2.pdf]

Supplemental Figure 2

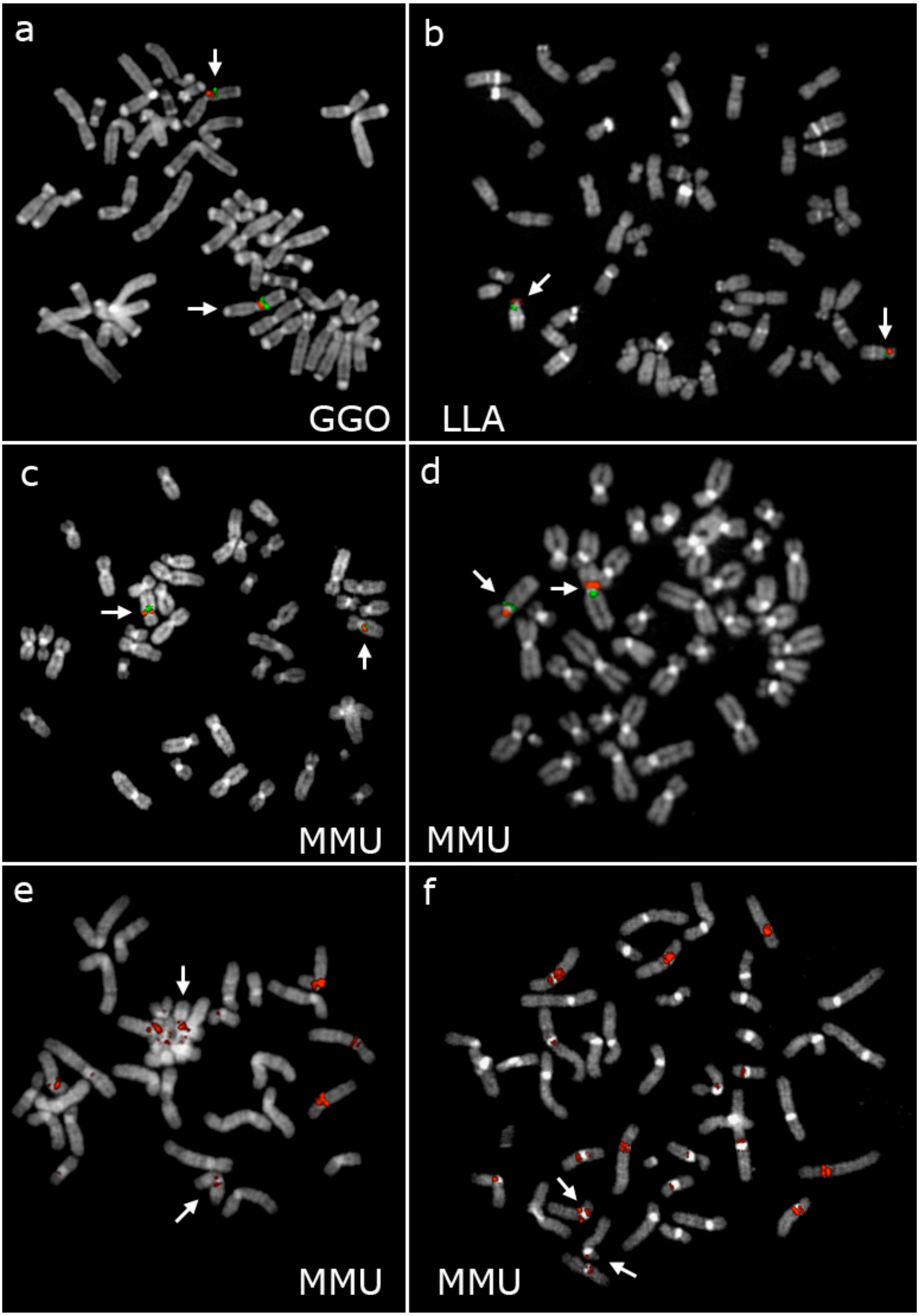

Examples of FISH experiments

(a), (b), (c) Examples of FISH experiments using BAC clones RP11-11C20 (in red; chr8:52,776,800-52,920,509) and RP11-442A17 (in green; chr8:63,772,024-63,955,120), used to track the evolution of chromosome 8 in gorilla [GGO (a)], woolly monkey [LLA (b)], and macaque [MMU, (c)].

(d) FISH experiment using BAC clones RP11-449O23 (in red; chr3:164,054,852-164,221,000) and
RP11-418B12 (in green; chr3:164,539,721-164,707,127) defining the position of the neocentromere of MMU2 (HSA3).

(e) FISH results of BAC clone CH250-449K18 mapping in the pericentromeric region of MMU14 (HSA11), indicated by the arrows.

(f) FISH results of BAC clone CH250-417O7 mapping in the pericentromeric region of MMU13 (HSA2q), indicated by the arrows.
