# Supplementary material for: Evolutionary-new centromeres preferentially emerge within gene deserts
Source: Genome Biol. 2008 Dec 16;9(12):R173. doi: 10.1186/gb-2008-9-12-r173 (PMC2646277; doi:10.1186/gb-2008-9-12-r173)
Supplement: Additional data file 3 — Human probes used to track the evolutionary history of chromosome 8. [file gb-2008-9-12-r173-S3.doc]

| **Supplemental Table 1** | | | |  |  |
| --- | --- | --- | --- | --- | --- |
| Human probes used to track the evolutionary history of chromosome 8 | | | | | |
| Code | BAC | Acc. N. | map | | Position (hg17, March 2004) |
| A | RP11-18D5 | AC090135 | 8p23.3 | | chr8:381,182-484,890 |
| B | RP11-737E8 | ends | 8p23.1 | | chr8:11,580,455-11,789,912 |
| C | RP11- 10C8 | AC019270 | 8p22 | | chr8:13,210,538-13,365,494 |
| C1 | RP11-120K21 | BES | 8p21.2 | | chr8:25,830,685-25,965,011 |
| D | RP11-10D7 | AC013603 | 8p12 | | chr8:33,487,657-33,665,495 |
| E | RP11-262I23 | BES | 8p11.22 | | chr8:39,846,706-40,045,213 |
| centromere | |  |  | |  |
| F | RP11-11C20 | AC090186 | 8q11.23 | | chr8:52,776,787-52,920,537 |
| G | RP11-442A17 | AC087358 | 8q12.3 | | chr8:63,772,024-63,955,120 |
| H | RP11-382J12 | AC022731 | 8q13.3 | | chr8:71,614,507-71,778,503 |
| I | RP11-91P17 | AC084706 | 8q21.12 | | chr8:79,158,450-79,305,261 |
| J | RP11-353O11 | AC091184 | 8q21.3 | | chr8:90,077,451-90,220,326 |
| K | RP11-410L14 | AC104986 | 8q22.2 | | chr8:99,944,884-100,098,300 |
| L | RP11-697C18 | AC024996 | 8q23.3 | | chr8:113,395,877-113,573,740 |
| M | RP11-414D17 | AC013545 | 8q24.12 | | chr8:122,296,267-122,496,452 |
| N | RP11-269I24 | AC090987 | 8q24.21 | | chr8:131,641,435-131,795,238 |
| O | RP4-698E23 | AF186192 | 8q24.3 | | chr8:145,807,985-145,953,950 |
| telomere | |  |  | | chr8:146,274,826 |

Human probes used to track the evolutionary history of chromosome 8.
