# Supplementary material for: Evolutionary-new centromeres preferentially emerge within gene deserts
Source: Genome Biol. 2008 Dec 16;9(12):R173. doi: 10.1186/gb-2008-9-12-r173 (PMC2646277; doi:10.1186/gb-2008-9-12-r173)
Supplement: Additional data file 4 — Species-specific BAC clones used in FISH experiments to detect pericentromeric segmental duplications. [file gb-2008-9-12-r173-S4.doc]

| **Supplemental Table 2** | | |  |  |
| --- | --- | --- | --- | --- |
| Species-specific BACs tested in FISH experiments to detect segmental duplications | | | | |
| ENC | BAC | | Position in HSA (May2004) | FISH result |
| MMU2 (HSA3) | | CH250-360I3 | chr3:163,068,376-163,231,644 | 2pcen |
|  | | CH250-450J9 | chr3:163,308,656-163,461,404 | 2pcen |
|  | | CH250-391E24 | chr3:163,861,235-164,012,006 | 2pcen |
|  | | CH250-3M19 | chr3:164,476,043-repeat | 2qcen |
|  | | CH250-22O9 | chr3:164,478,437-164,626,209 | 2qcen |
|  | | CH250-168E6 | chr3:164,482,714-repeat | 2qcen |
|  | | CH250-412C22 | chr3:164,574,295-repeat | 2qcen |
|  | | CH250-154L6 | chr3:164,584,933-repeat | 2qcen |
|  | | CH250-147E24 | chr3:164,584,915-164,702,020 | 2qcen |
|  | | CH250-154F3***** | chr3:164,615,766-alphoid | 2qcen |
|  | | CH250-366G12 | chr3:164,651,157-164,786,709 | 2qcen |
|  | | CH250-146H16 | chr3:164,774,603-164,933,307 | 2qcen |
|  | | CH250-91J4 | chr3:164,777,358-164,967,209 | 2qcen |
|  | | CH250-25G4 | chr3:164,777,358-164,967,209 | 2qcen |
| MMU12 (HSA2q) | | **CH250-359C1** | chr2:138,344,201-138,510,183 | 12pcen+12qcen |
|  | | **CH250-158G21** | chr2:138,478,651-138,621,067 | 12pcen+12qcen |
|  | | **CH250-18F12*** | chr2:138,643,711-alphoid | 12pcen+12qcen+multiple signals pericentromeric regions |
| MMU13 (HSA2p) | | CH250-405M2***** | chr2:86,752,837-alphoid | 13qcen |
|  | | **CH250-565F19*** | chr2:86,755,212-alphoid | 13pcen+13qcen+multiple signals pericentromeric regions |
|  | | CH250-323I4 | chr2:86,772,302-repeat | 13qcen |
|  | | **CH250-417O7** | chr2:86,785,727-repeat | 13pcen+13qcen+multiple signals pericentromeric regions |
|  | | **CH250-371E19*** | chr2:86,870,586-alphoid | 13pcen+13qcen+multiple signals pericentromeric regions |
|  | | CH250-413J11 | chr2:86,870,149-88,154,238 | 13qcen |
| NLE15 (HSA11) | | CH271-107P22 | chr11:89,564,839-89,688,390 | 15pcen |
|  | | **CH271-140J13** | chr11:89,572,864-repeat | 15pcen+15qcen |
|  | | CH271-171J24 | chr11:89,527,557-89,683,905 | 15pcen |
|  | | CH271-207G22 | chr11:89,627,320-89,736,814 | 15pcen+15q (at the level of dup)+multiple signals |
|  | | CH271-238J17 | chr11:89,737,813-repeat | 15pcen+15q (at the level of dup)+multiple signals |
|  | | CH271-2N18 | chr11:89,654,287-89,804,787 | 15pcen+15q (at the level of dup)+multiple signals |
|  | | CH271-3I16 | chr11:89,664,517-repeat | 15pcen+15q (at the level of dup)+multiple signals |
|  | | CH271-72L03 | chr11:89,648,224-89,804,808 | 15pcen+15q (at the level of dup)+multiple signals |
|  | | CH271-72L13 | chr11:89,726,139-89,883,920 | 15pcen |
| MMU15 (HSA9) | | CH250-173A19 | chr9:122,145,901-repeat | 15q cen |
|  | | **CH250-221O11*** | chr9:122,220,400-alphoid | 15pcen+15qcen+multiple signals pericentromeric regions |
| MMU17 (HSA13) | | **CH250-310C22** | chr13:61,479,136-61,591,608 | 17qcen+17pcen |
|  | | **CH250-299M13** | chr13:61,503,914-61,617,441 | 17qcen+17pcen |
|  | | **CH250-115C9** | chr13:61,540,997-61,676,877 | 17qcen+17pcen+X peric |
| MMU18 (HSA18) | | CH250-223P11 | chr18:50,033,356-50,202,866 | 18pcen |
|  | | CH250-548K3 | chr18:50,037,439-50,201,647 | 18pcen |
|  | | CH250-254K12 | chr18:50,361,150-repeat | 18pcen |
|  | | CH250-185G6 | chr18:50,397,594-50,527,863 | 18pcen |
|  | | CH250-545B4 | chr18:50,399,406-repeat | 18pcen |
|  | | CH250-157J11 | chr18:50,400,737-50,572,372 | 18pcen |
|  | | CH250-549O6 | chr18:50,415,300-repeat | 18pcen |
|  | | **CH250-322J6** | chr18:50,437,322-repeat | 18pcen+18 qcen |
|  | | CH250-568D23 | chr18:50,501,290-50,501,290 | 18pcen |
|  | | CH250-573K15 | chr18:50,504,330-50,656,840 | 18pcen |
|  | | CH250-215N18 | chr18:50,514,852-repeat | 18pcen |
| MMU14 (HSA11) | | CH250-153H2 | chr11:5,737,949-repeat | 14qcen |
|  | | **CH250-444O7*** | chr11:5,861,684-alphoid | 14pcen +multiple signals pericentromeric regions |
|  | | CH250-240L19***** | chr11:5,879,925-alphoid | 14pcen |
|  | | **CH250-499K18*** | chr11:6,038,164-alphoid | 14pcen+14qcen +multiple signals pericentromeric regions |

List of all species-specific BAC clones, mapping in the pericentromeric regions of ENCs, tested in FISH experiments. BACs in bold detected segmental duplications (selectively reported in Table 3). One BAC-end of asterisked BACs is entirely composed of alphoid repeats.
